# Trace elements and carcinogenicity: a subject in review

**DOI:** 10.1007/s13205-012-0072-6

**Published:** 2012-06-10

**Authors:** Stephen Juma Mulware

**Affiliations:** Ion Beam Modification and Analysis Laboratory, Department of Physics, University of North Texas, 1155 Union Circle, #311427, Denton, TX 76203 USA

**Keywords:** Carcinogenicity, Trace elements, Epidemiology, ROS, Exposure

## Abstract

Cancer is known to be a multi-step process, which involves different stages including initiation, promotion, progression and metastasis. Chemical carcinogens including most trace elements can change any of these processes to induce their carcinogenic effects. Various studies confirm that cancer arises from the accumulation of irreversible DNA damage, which results from multiple mutations in critical genes in the body organ. Chemical carcinogens most often directly or after xenobiotic metabolism, act as genotoxic causes to induce DNA damage. Genotoxic carcinogen refers to a group of chemicals capable of producing cancer by directly altering the genetic material of target cells. Other carcinogens are however classified as non-genotoxic, which represents chemicals that are capable of producing cancer by some secondary mechanism not related to direct gene damage. They act as tumor promoters, endocrine-modifiers, receptor mediators, immunosuppressant, or inducers of tissue-specific toxicity and inflammatory responses. The diversity of modes of action, of non-genotoxic carcinogens, the tissue and species specificity and the absence of genotoxicity makes it extremely hard to predict their carcinogenic potential. The roles of trace metals (some of which are either genotoxic or non-genotoxic) in cancer development and inhibition have a complex character and have raised many questions because of their essential and toxic effects on people’s health. Trace metals such as cadmium, nickel, arsenic, beryllium and chromium (VI) have been recognized as human or animal carcinogens by International Agency for Research on Cancer (IARC). The Carcinogenic capability of these metals depends mainly on factors such as oxidation states and chemical structures. The oxidative concept in metal carcinogenesis proposes that complexes formed by these metals, in vivo, in the vicinity of DNA, catalyze redox reactions, which in turn oxidize DNA. The most significant effect of reactive oxygen species in the carcinogenesis progression is DNA damage, which results in DNA lesions like strand breaks and the sister-chromatid exchange. This article reviews the carcinogenicity of various trace elements.

## Introduction

Carcinogenic substances are those that induce tumors (benign or malignant), increase their incidence or malignancy or shorten the time of tumor occurrence when they get into the body through inhalation, injection, dermal application or ingestion (Maurici et al. 2005, p. 177). Carcinogens are classified as either genotoxic or non-genotoxic depending on their modes of action. Genotoxic carcinogens are those which initiate carcinogenesis by direct interaction with DNA, resulting in DNA damage or chromosomal aberrations that can be detected by genotoxicity tests (OECD 2006). On the other hand, non-genotoxic carcinogens are agents that indirectly interact with the DNA, causing indirect modification to DNA structure, amount, or function that may result in altered gene expression or signal transduction (OECD 2006). Substances that induce tumors in animals are also considered human carcinogens until proven otherwise (UNECE 2004, p. 167). All known human carcinogens that have been evaluated adequately in animal bioassays have been found to be also carcinogenic in animal bioassay studies. In fact, it has been reported that of the nearly 100 known genotoxic and non-genotoxic human carcinogens, one-third were shown first to be carcinogenic in animals (Huff 1999a, b). Other studies have demonstrated a strong correlation between carcinogenic potencies estimated from epidemiological data and those from animal carcinogenesis bioassays (Allen et al. 1988; Goodman and Wilson 1991). These observations have been used as guidelines to avoid human exposure to such chemicals found to be carcinogenic in laboratory animals.

According to evaluations done by the International Agency for Research on Cancer (IARC), carcinogenicity data reviewed of various trace elements are classified as: (i) sufficient, when a casual association is established between exposure to an agent and human cancer; (ii) limited, when an association has been observed but chance, bias, and confounding cannot be ruled out and (iii) inadequate, when the data are of insufficient quality, consistency or statistical power to allow a conclusion (IARC 1989). In the IARC evaluations, carcinogenicity of chemicals is categorized according to the following groups:

- Group 1: Carcinogens to humans, when the evidence is sufficient (105 agents). These comprise 61 chemicals, 9 viruses or pathogens (i.e. HIV), 16 mixtures (i.e. coal-tars) and 19 exposure circumstances (i.e. chimney sweeping).
- Group 2A: Probably carcinogenic to humans, mainly for experimental carcinogens with limited data to humans (66 agents). These comprise 50 chemicals, 2 viruses or pathogens, 7 mixtures and 7 exposure circumstances.
- Group2B: Possibly carcinogenic to humans, mainly for experimental carcinogens with less than limited evidence from humans and less than sufficient evidence from animals (248 agents). These comprise 224 chemicals 4 viruses or pathogens, 13 mixtures and 7 exposure circumstances.
- Group 3: Not classifiable as to its carcinogenicity to humans, for agents that do not fall into any other category (515 agents). These comprise 496 chemicals, 11 mixtures and 8 exposure circumstances.

Speciation remains the main methodological method problem of data review on the carcinogenicity of trace elements. Just a few compounds within the family (classification) have available data in most cases, and these compounds may sometimes not be the most relevant with regards to human exposure. For instance, epidemiological studies on occupationally exposed persons may not give conclusive effects to exposure to individual chemicals within the family. This is confounded by the fact that the effect of non-occupational exposures like smoking is rarely taken into consideration.

The questionable relevance of the route of exposure (e.g. subcutaneous or intramuscular administration) in animal experiments makes the use of data derived from such experiments problematic. The degree of solubility of chemical exposure, which influences biological effects as well as the long- or short-term experimental studies must be taken into account while deciding carcinogenicity classifications. Certain trace elements like zinc and selenium have been found to have anti-carcinogenic effect where as others tend to be carcinogenic in specific organs while showing no such effect in certain organs. No formal evaluation of anti-carcinogenic effects of these trace elements has been made by IARC. Table 1 below shows the summary of the IARC evaluations of the carcinogenicity of trace elements and other related compounds as detailed in the preamble of the volumes of the IARC monograph series (IARC 1993).

**Table 1:** IARC evaluations made of the carcinogenicity of trace elements and related compounds to humans by the international agency of research on cancer (IARC 1993)

Group 1: carcinogenic to humans		Group 2A: probably carcinogenic		Group 2B: possibly carcinogenic		Group 3: not classifiable	
Trace element	Chemical group	Trace element	Chemical group	Trace element	Chemical group	Trace element	Chemical group
As	Arsenic and its compounds	Pl	Cisplatin	Sb	Antimony trioxide	Sb	Antimony trisulfide
Be	Beryllium and its compounds	–	–	Co	Cobalt and its compounds	Cr	Metallic and trivalent chromium compounds
Cd	Cadmium and its compounds	–	–	Pb	Inorganic lead compounds	Fl	Inorganic fluorides
Cr	Hexavalent chromium compounds	–	–	Ni	Metallic nickel	Fe	Ferric oxide and hematite
Ni	Nickel compounds	–	–	–	–	Pb	Organic lead compounds
–	–	–	–	–	–	Hg	Metallic and inorganic mercury compounds
–	–	–	–	–	–	Se	Selenium and its compounds
–	–	–	–	–	–	Ti	Titanium dioxide

The carcinogenic capability of trace metals depends mainly on factors such as oxidation states and chemical structures. The oxidative concept in metal carcinogenesis signifies that complexes formed by these metals, in vivo, in the vicinity of DNA, catalyze redox reactions, which in turn oxidize DNA. The most significant effect of reactive oxygen species (ROS) in the carcinogenesis progression is DNA damage, which results in DNA lesions like strand breaks and the sister-chromatid exchange (Valco et al. 2005). It has been estimated that approximately 2 × 10^4^ DNA damaging events occur in every cell per day (Ames and Shigenaga 1992); a major portion of these occur via ROS. Similarly, ROS damage results in lipid peroxidation and depletion of protein sulfhydryls. Even though the increase in oxidative DNA lesions has been frequently attributed to metal exposures, it is important to note that the molecular mechanism leading to tumor formation after such exposures is still not well understood. The metal carcinogenesis is mediated either by the increased generation of ROS on the basis of ESR spin trapping studies or by interference with the repair process of DNA (Mehmet 2006). Some oxygen species are worst carcinogenic molecules. There is a very fine balance between enzymatic [such as superoxide dismutase (SOD), glutathione peroxidase and catalase] and non-enzymatic (such as ascorbic acid, α-tocopherol, β-carotene and isoflavons) antioxidants and free radicals in each cell. When ROS production is higher than the cell reduction capabilities, they can induce lipid peroxidation, depletion of the sulfhydryl groups, change signal transduction pathways, calcium homeostasis and DNA damage (Valco et al. 2005). This may result in occurrence of aging effect and cancer infection.

Free radicals that contain ROS are chemical compounds that posses one or more electrons in their outer orbital, while non-radical species have the electron pairs with anti-parallel spin. The electron transfer reaction in the cell is responsible for the production of free radicals. Superoxide anion (O_2_^·−^), which occurs about 2–5 % by transformation of O_2_ consumed by mitochondria and endoplasmic reticulum, is one of the major radical species. The other major radical species includes hydrogen peroxide (H_2_O_2_), which is generated spontaneously via reaction between superoxide anion and hydrogen cation, by means of SOD in biological systems (Augusto and Rhian 2011). Other radicals includes: the highly reactive hydroxyl radical (OH·) and peroxyl radicals (ROO^·−^), hyperchloride (HOCl), alkoxyl radical (RO·), thiyl radical (RS·) and nitric oxide (NO). The ROS occurring in a given cell depends on the metal species: For example, arsenic-mediated generation of ROS involves generation of superoxide (O_2_^·−^), peroxyl radical (ROO·), nitric oxide (NO·), hydrogen peroxide (H_2_O_2_) and dimethylarsinic peroxyl radical ((CH_3_)_2_AsOO·). In the absence of the metal catalysts, superoxide and hydrogen peroxide can be briefly ineffective (Mehmet 2006). Consequently, the protective enzymes such as SOD, catalase and glutathione peroxidase can moderate the effects of ROS.

It is important to note that despite their toxic effects, trace metals in right proportion and concentration, are essential to normal human homeostasis. The four main functions of essential trace metals include: (i) stabilizers, (ii) elements of structure, (iii) essential elements for hormonal function and (iv) co-factors in enzymes. The cell structure alone or structural function due to lack of stabilization, change of charge properties and allosteric configuration can be affected by inadequate supply or lack of essential trace elements (Mehmet 2006). Some trace elements play a major role as enzyme co-factors, for instance SOD are a group of metallo-enzymes (containing Fe, Mn, or Cu and Zn) that catalyze the disproportionate of superoxide free radical (O_2_^·−^) to form hydrogen peroxide and dioxygen. These enzymes constitute the defense system against the cytotoxic superoxide free radical. Cu, Zn-SOD, which is a prototypical dinuclear metalloprotein, is an important antioxidant enzyme for cellular protection from ROS and several proteins involved in DNA repair (Auld 2001; Ho 2004). Metallothionein (MT) is a transition metal-binding protein that is found in all eukaryotes (cells with nucleus) and in some prokaryotes. MT plays a role in the homeostasis of essential metals such as zinc and copper, detoxification of toxic metals such as cadmium, and protection against oxidative stress. Due to MT’s important role in transcription factor regulation, problems with MT function or expression may lead to malignant transformation of cells and even cancer. Copper plays a role in activation of more than 30 enzymes which includes caeruloplasmine, cytochrome oxidase, lysine oxidase, dopamine-hydroxylase, ascorbate oxidase and tyrosinase among others in the body, some of which is involved in the synthesis of the main component of connective tissues called collagen. Iron too plays a significant role in oxygen transportation, xenobiotic metabolism, oxidative phosphorylation and act as prosthetic group in many enzymes (Anil et al. 2011).

The purpose of this review is to discuss the carcinogenic role of trace elements and the possibility of using their assay in biological fluids for diagnostic or prognostic purposes in cancer patients. The review will look into experimental results of previous works and discuss the possibility of variations in trace elemental concentrations obtained.

## Carcinogenicity of trace elements

### Arsenic and arsenic compounds

Arsenic occurs mostly as a metal arsenide in sulfur-containing ores. It is distributed widely in natural waters but in some locations it is added to the environment through the use of certain insecticides and the combustion of fossil fuels. In the natural waters, arsenic occurs in oxidation states III and V in form of arsenous acid (H_3_AsO_3_) and arsenic acid (H_3_AsO_5_) and their salts, respectively (Sawyer et al. 2003). As determined by IARC report, arsenic and its compounds are highly carcinogenic to humans. Skin cancer and lung cancer have been reported in patients treated with inorganic trivalent arsenic compounds, drinking water with high levels of arsenic and those with occupational exposures to inorganic arsenic compounds in mining and copper smelting industries, respectively (Boffetta 1993). Other cancers liked to arsenic toxicity include kidney and bladder (ATSDR 2003). The toxicity of arsenic and its inorganic compounds has been classified as: acute toxicity, sub-chronic toxicity, genetic toxicity, developmental and reproductive toxicity (Chakraborti et al. 2004); immunotoxicity (Sakurai et al. 2004); biochemical and cellular toxicity (Mudhoo et al. 2001). The toxicity of inorganic arsenic can induce oxidative stress leading to the inhabitation of DNA repair. It has been reported that arsenic-induced oxidative stress also causes DNA strand breaks, alkali-labile sites, which eventually results into DNA adducts (Pu et al. 2007). Arsenic mediation can also alter methylation status of oncogenes and tumor suppressor genes and in the processes enhancing carcinogenesis (Reichard and Puga 2010).

Drinking contaminated ground water with elevated arsenic levels is the main source of exposure to humans across the globe, but in particular in South Asia. The resulting widespread carcinogenic effects on human health led to the recommendation by USEPA to reduce the drinking water maximum contaminant limit to 0.010 mg/L in line with the WHO guidelines (USEPA 2005). In Asian countries, arsenic compounds have been introduced in the food chain through uptake by rice plans in the irrigation fields. However, the major source of arsenic in human food is the fish and seafood. It was reported that there was a link between pancreatic cancer and arsenic exposure among children who consumed arsenic contaminated powdered milk (Yurifuji et al. 2011). Other studies have shown that the organic form of arsenic in the food substances is not as much harmful as the inorganic forms ingested in drinking waters. Organo-arsenic intake of about 0.05 mg/kg body weight was found to have less toxic effect (Uneyama et al. 2007).

A systematic assessment of cofactors associated with increased arsenic-induced cancer risk has been done. It was established that arsenical skin lesions (i.e., melanosis, leucomelanosis and keratosis) are a hallmark of chronic arsenic toxicity. These skin lesions may appear within a few years of exposure unlike other arsenic-induced cancers, which have a longer latency (i.e., decades). These studies show that the risk of arsenic-induced skin lesions and cancers is greater among men than in women. Other variables including cigarette smoking and ultraviolet radiation have been identified as independent risk factors of non-melanoma skin cancer, and contributors to the higher risk of arsenic-related skin lesions in men. In addition, occupational exposures, nutritional habits, socioeconomic status, and genetic factors, have also been shown to influence the of arsenic exposure effects on skin lesions and cancer (Ahsan et al. 2003a, b; Chen et al. 2006).

### Beryllium and beryllium compounds

Beryllium (Be) occurs naturally in the form of beryllium aluminum silicate (beryl), 3BeO·Al_2_O_3_·6SiO_2_. Its naturally present in rocks, coal and oil, soil and volcanic dust (ATSDR 2008).There are over 40 known beryllium-bearing minerals, however, only beryl and bertrandite (4BeO·2SiO_2_·H_2_O) are commercially vital. Beryl contains ca. 11 % beryllium oxide while Bertrandite contains less than 1 % beryllium, which can be processed into beryllium hydroxide. Beryllium forms alloys with many metals making them much stronger, with high electrical and thermal conductivity as well as high resistance to corrosion and fatigue. These properties make beryllium an important component of wide range of industrial applications including electronics and weaponry among others.

Occupational exposure to beryllium had been shown to contribute to an upward trend and reported deaths from lung cancer developed by those who had had beryllium-induced respiratory distress. Liver cancer and pancreatic cancer was also reported to be high among those exposed (Stoeckle and Mancuso 1974). According to IARC reports, beryllium inhalation by experimental animals, intratracheal studies on rats, intrabronchial studies on monkeys and intravenous or intramedullary administration to rabbits showed sufficient evidence of carcinogenicity to beryllium and its compounds (Boffetta 1993). The increased risk of carcinogenesis to the exposure to beryllium and beryllium compounds made it to be classified as one of the most carcinogens (group 1) to human by IARC.

The binding of ionic beryllium to nucleic acids cause genetic transformation in mammalian cells which may result into infidelity of DNA replication (Leonard and Lauwerys 1987). This genetic transformation is likely to cause lung cancer to the exposed individuals. A high relative risk of up to 2.3 would likely cause acute beryllium pneumonitis, which is linked to higher lung cancer rates (Steenland and Ward 1991). The same study has shown that the probability of contracting lung cancer is increased among those with acute beryllium disease (standard mortality ratio, SMR = 2.32) than in those with chronic beryllium disease (CBD) (SMR = 1.57) (Steenland and Ward 1991). A report on carcinogens indicated that the exposure to beryllium and its compounds caused the growth of lung tumors in rats that were exposed through a single intratracheal instillation or a 1-h inhalation (Second Annual Report 1981). The same report also stated that monkeys exposed to beryllium oxide and beryllium sulfate through intrabronchial implantation or inhalation developed anaplastic carcinoma where as rabbits exposed to beryllium metal, beryllium carbonate, beryllium oxide, beryllium phosphate, beryllium silicate or zinc beryllium silicate by intravenous injection and/or bone implantation developed osteosarcoma (Second Annual Report 1981).

The immunological behavior of T lymphocytes (T cells, which play a central role in cell-mediated immunity) following beryllium exposure has been explained in terms of toxic kinetic and toxic dynamic effects, for instance, 70 % of the total beryllium, that is, 10–100 ng/g serum was detected in pre-albumin and clinical studies of acute beryllium disease, respectively (Stiefel et al. 1980; Cooper and Harrison 2009). The programmed death pathway (PD-1) is active in beryllium-induced disease and significantly control beryllium-induced T cell proliferation (Palmer et al. 2008). Studies have shown that beryllium exposure in the cell mediates a thiol imbalance that leads to oxidative stress, which in turn may transform the proliferation and clonal expansion of CD4+ T cells (Dobis et al. 2008). Beryllium-induced proliferation of T lymphocytes from the lungs of a CBD patients are associated with human leukocyte antigens (HLA-DP) alleles which posses a glutamic acid in the 69th position of the B1 chain of this molecule (Fontenot et al. 1999; Wang et al. 2001). It has been found that exposure to beryllium-ferritin cause the production of persistent antigen, which is linked to the chronicity of CBD and its development even years after one had been exposed (Cooper and Harrison 2009). Since the presence of circulating beryllium-specific CD4+ T cells directly correlates with the severity of lymphocytic alveolitis, studies show that there is a great increase in antigen-specific effecter memory CD4+ T cells in the lungs of CBD patients (Fontenot et al. 2002).

### Cadmium and cadmium compounds

Cadmium is naturally present in air, soils sediments and even unpolluted sea water. It is emitted to the air by mines, metal smelters and industries, which use cadmium compounds for alloys, batteries, pigments and plastics (Harrison 2001). It is a widespread environmental and industrial pollutant, which has been declared carcinogen by International Agency for Cancer Research (IARC 1993). All forms of tobacco contains an appreciable amount of cadmium making tobacco smoking, both active and passive, the largest single source of human exposure to cadmium and in effect its carcinogenic effect. The smoking effects are pronounced by the fact that the absorption of cadmium from the lungs into the body cells is much greater than the gastrointestinal tract absorption (Figueroa 2008; Ming-Ho 2005). Since cadmium ions are readily absorbed by plants and stored in the stem, leaves and fruits, which animals can feed on, affected animal and plant food products also acts as the sources of cadmium to humans. Only inorganic cadmium salts are present in food products. Cadmium accumulation has been reported in milk and animal fatty tissues (Figueroa 2008) and sea foods such as mollusks and crustaceans (Castro-González and Méndez-Armenta 2008; WHO 2004b, 2006). EPA recommendation for maximum contaminant level of cadmium in drinking water is 0.005 mg/L while recommended a lower level of 0.003 mg/L (WHO 2004a).

Animal-based studies have reported carcinogenic effects from cadmium exposure. Neoplasms in rats were reported following subcutaneous, intramuscular and intraprostatic cadmium injection, while respiratory tumors in rats developed from inhalation. Occupational exposures of workers in battery and metal smelting industries have been linked to prostate, kidney and lung cancers, however smoking could have been a factor in these studies as well (Nordberg et al. 1992). Another study on trace elemental analysis of carcinoma kidney and stomach also reported an elevated cadmium level in the cancer tissue of kidney than the in the normal (Reddy et al. 2003). Studies have shown that cadmium have no essential functions in human body and can produce toxic effects even at very low dose (Binner et al. 1983). This toxicity results from both extracellular and intracellular interaction between cadmium and the biological system. Cadmium acts as an inhibitor of sulfydryl enzymes and also has got affinity for other cell ligands like hydroxyl, carboxyl, phosphatyl, cysteinyl and hystidyl side chains of protein, and as such can disrupt pathways of oxidative phosphorylation (Dara 1997). Since there is no homeostatic control of cadmium in the human body, it is highly toxic trace element. The target organ which is the kidney stores more than a third of all absorbed cadmium in the body. When high amounts of Cd^2+^ are introduced into the body, it replaces Zn^2+^ at the key enzyme sites causing metabolic disorders. Other toxic effects include respiratory disorders, damage to the kidney and development of kidney stones.

The carcinogenic activity of cadmium varies in different studies. While some in vitro studies have shown that cadmium induces DNA strand breaks, DNA–protein cross-links, oxidative DNA damage and chromosomal aberrations, mostly at high cytotoxic concentrations (Mouron et al. 2001). Other studies, however, show that it modulate the expression of pro-oncogenes (Waisberg et al. 2003), and inhibit the function or expression of the DNA repair systems (Giaginis et al. 2006). While the main ROS responsible for oxidative DNA damage in cells appears to be hydroxyl radicals generated by the reaction of reactive transition metal ions, such as Fe^2+^ and Cu^2+^ with H_2_O_2_ (Henle and Linn 1997), cadmium is not redox active but contributes indirectly to oxidative stress by affecting the cellular thiol redox balance (Nzengue et al. 2008). Other studies have shown that cadmium indirectly generates ROS and consequently DNA, lipid and protein oxidation in various cell lines, which lead to DNA damage and hence tumor growth (Wang et al. 2004).

### Chromium and chromium compounds

Chromium is a naturally occurring element in rocks, animals, plants, soil and volcanic dust and gases. Chromium predominantly occurs in the environment in one of two valence states: trivalent chromium (Cr III), which occurs naturally and is an essential dietary nutrient needed for to normal glucose, protein and fat metabolism, and hexavalent chromium (Cr VI), which, along with the less common metallic chromium (Cr 0), is most often produced by industrial processes (ATSDR 1998). Ferrochrome production industries including ore refining, chemical and refractory processing, cement-producing plants, automobile brake lining and catalytic converters for automobiles, leather tanneries and chrome pigments are mostly linked to the atmospheric burden of chromium (USEPA 1998). Humans are exposed to chromium (generally chromium [III]) by eating food, drinking water and inhaling air that contains the chemical with the average daily intake from air, water and food estimated to be less than 0.2–0.4, 2.0 and 60 μg, respectively (ATSDR 1998). Dermal exposure to chromium may occur during the use of consumer products that contain chromium, such as wood treated with copper dichromate or leather tanned with chromic sulfate can lead to dermal exposure.

There is little evidence for the carcinogenicity of metallic chromium and trivalent chromium compounds in humans. Cr(III) is actually an essential human nutrient required to promote the insulin action for utilization of sugars, proteins and fats. It increases the insulin binding to cells, insulin receptor number and also activates the insulin receptor kinase which in effect increases insulin sensitivity. However, there is sufficient evidence that hexavalent chromium compounds are carcinogenetic. These compounds include: calcium chromate, zinc chromate, strontium chromate and lead chromate. Occupational exposure to these hexavalent chromium compounds in chromate production, chromate pigment production and chromium plating industries have been linked to the cancer of the lung, nose and nasal sinuses (Boffetta 1993).

Chromium ions is most carcinogenic in the form of , which enters the body cell by sulfate uptake pathway and is ultimately reduced to Cr(III) through a Cr(IV)-glutathione intermediate species. The hexavalent latter complex then binds with the DNA to produce a kinetically inert and potentially damaging lesion and can cause abnormal phenotype due to the formation of ROS (Bertini et al. 1998). Chromium is also believed to repress p53, a suppressor protein whose inactivation through mutations is associated with many types of human cancers. The p53 protein is essential for many biological processes including regulation of genes involved in cell cycle, cell growth arrest after DNA damage and apoptosis (Hollstein et al. 1994). The reactive chromium intermediates like Cr(V) and Cr(IV) generate OH· radicals which cause DNA strand breaks, base modification, lipid peroxidation and nuclear transcription factor NF-kB activation. This also inactivates p53which alters the process of p53-dependent cell cycle arrest and apoptosis eventually enhancing cancer development (Wang et al. 2000). The unstable intermediate Cr(V) and Cr(IV) ions also activates oxygen agents and thiyl and organic radicals (RS· and R·), which are responsible for the DNA and other observed cellular damage.

### Cobalt and cobalt compounds

Most of the Earth’s natural cobalt is located in its core. Cobalt exists in relatively low abundance in the Earth’s crust and in natural waters, from which it is precipitated as the highly insoluble cobalt sulfine (CoS). The soil content of cobalt ranges from 0.1 to 70 ppm in other areas with an average of about 8 ppm. The blue green algae (cyanobacteria) in marine environments and other nitrogen fixing organisms use cobalt. Cobalt is not found as a free metal and is generally found in the form of ores. Cobalt is usually not mined as a free metal but rather in ores usually as a by-product of nickel and copper mining activities with its main ores being cobaltite, erythrite, glaucodot and skutterudite. Human exposure occurs through inhalation of cobalt in air, drinking water and eating food that contains cobalt. Skin contact with soil or water that contains cobalt may also be an exposure agent. Plants uptake may also increase accumulation of cobalt in plants and hence animals that feed on them. Cobalt is essential for human health since it is a part of vitamin B_12_. Due to its ability to stimulate red blood cells, cobalt is also used in the treatment of anemia in pregnant women. However, occupational exposure to high concentrations of cobalt may have led to lung effects, such as asthma and pneumonia.

The International Agency for Research on Cancer (IARC) has listed cobalt and cobalt compounds within group 2B (agents which are possibly carcinogenic to humans). Metallic cobalt and bivalent cobalt oxide was found to be carcinogenic in experimental animals (rats) at a relatively high dose, by route(s), histologic type(s) or by mechanism(s) that are not considered relevant to worker exposure. However, other cobalt compounds including bivalent cobalt sulfide, and bivalent cobalt chloride had limited carcinogenic evidence in experimental animals after subcutaneous or intramuscular administration (Boffetta 1993). Bivalent and trivalent cobalt oxide, cobalt nephthenate and trivalent cobalt acetate had shown insufficient evidence of carcinogenicity in experimental animals. Humans with implants of cobalt containing prostheses reported increased risk of sarcomas and blood neoplasms. Occupational exposure epidemiological studies reported increased risk of lung cancer (IARC 1991).

Studies have shown that in the presence of hydrogen peroxide, micromolar concentrations of cobalt (II) ions can cause chemical damage to DNA bases in chromatin, probably through the production of hydroxyl radicals in vitro in human lymphocytes and isolated DNA (Lloyd et al. 1997; Nackerdien et al. 1991). The ROS generation by cobalt (II) from H_2_O_2_ and related in vitro DNA damage was investigated with electron spin resonance, electrophoretic assays, and high performance liquid chromatography. The same study showed that the oxidation potential of cobalt (II) can be modulated by chelators to facilitate or reduce its capacity to generate ROS from hydrogen peroxide (H_2_O_2_), for instance, the study found that anserine enhanced the capacity of cobalt (II) to generate radicals, while deferoxamine reduced it (Mao et al. 1996). Other studies have shown that cobalt ions substitute for zinc in protein–zinc finger domains, which control the transcription of several genes generate free radicals close to DNA, causing DNA damage in the process while others indicate that cobalt ions affect the repair of DNA damage induced by other agents in mammalian cells (Sarkar 1995; Hartwig et al. 1991).

### Lead and inorganic lead compounds

Lead occurs naturally as a pure metal found in small amounts in the earth’s crust. Lead can also be found in plants, animals, air, water, dust and soil. Lead exists both as a free metal and also in various compounds. In organic lead compounds, such as tetraethyl lead and tetramethyl lead, are combined with carbon groups while lead oxide and lead chloride, are combinations of lead with other elements. Human exposure is mainly by inhalation from dust or fumes and by swallowing. Due to its widespread uses, more environmental exposures are from man-made rather than natural sources.

Inorganic lead salts, with ionic bonds like lead acetate, have different chemical properties and toxicological effects compared to organolead compounds, with covalent bonds like tetraethyl lead. Several epidemiological and animal experimental works suggest that inorganic lead compounds are associated with increased risks of tumorigenesis. Lead acetate, lead sub-acetate and lead phosphate have been found to cause tumors in the kidneys of rats and mice (IARC 1997). In rats, the carcinogenic risks of lead compounds can be induced at doses that are not associated with organ toxicity and in mice that do not produce α-2 urinary globulin in the kidney. The kidney, particularly the tubule epithelium of the renal cortex, is the principal target organ for the carcinogenicity of lead salts in animals, irrespective of the route of administration. Since renal epithelial tumors are very rare in untreated rats and mice of both sexes, even low incidences of these neoplasms in lead treated animals are good indicators of a carcinogenic effect (IARC 1980, 1987).

Most observed mechanisms of lead carcinogenicity include direct DNA damage, clastogenicity, or inhibition of DNA synthesis or repair (Landolph 1994; Hartwig et al. 1990). Lead can also cause the generation of ROS and cause oxidative damage to DNA. Other studies have shown that lead can substitute for zinc in several proteins that function as transcriptional regulators, including protamines (Quintanilla-Vega et al. 1997), hence reducing the binding of these proteins to the recognition elements in genomic DNA, a process which suggests an epigenetic involvement of lead in altered gene expression. These events may be of particular relevance in transplacental exposures and later cancer. Lead–zinc interactions in proteins can also cause post-translational changes in protein structure. The tumor suppressor protein p53 is a zinc-binding protein and thus if zinc is displaced by lead in p53, may result in a structurally altered form of the protein with functional consequences not different from mutation or deletion of the p53 gene as has been found for cadmium (Hainaut and Milner 1993). Occupational exposures have been linked to increase risk of lung stomach, kidney and bladder cancers in humans.

### Nickel and nickel compounds

Most nickel on Earth mainly found in the iron–nickel molten core, which is 10 % nickel. Nickel is easily absorbed by organic matter which explains why coal and oil contain considerable amounts. The soil’s nickel content ranges between 0.2 and 450 ppm in some clay and loamy soils with an average of about 20 ppm. Nickel occurs in some beans where it is an essential component of some enzymes and in tea which has 7.6 mg/kg of dried leaves.

Nickel occurs combined with sulfur in millerite, with arsenic in the mineral niccolite and with arsenic and sulfur in nickel glance. Most ores from which nickel is extracted are iron–nickel sulfides, such as pentlandite. Other sources of nickel include chocolate and fats, vegetables grown in contaminated soils as well as tobacco products. Humans are exposed to nickel through inhalation, drinking contaminated water, eating food or smoking cigarettes and skin contact with nickel-contaminated soil or water. In small quantities nickel is essential, but when the uptake is too high it can be a danger to human health.

Occupational exposure to nickel occurs in industries that include: high temperature oxidation of nickel matte and nickel–copper matte, electrolytic refiners, copper plants and during nickel salt extraction in the hydrometallurgical industry. Epidemiological studies among nickel refinery workers exposed to nickel sulfate and a combination of nickel sulfides and oxides showed high risk of nasal cancer, larynx cancer, prostate cancer and lung cancer (Boffetta 1993). The chromosomal damage caused by carcinogenic nickel compounds (crystalline nickel sulfide and nickel chloride) has been found to be consistent with their carcinogenicity and is affected by many factors, including the exposure concentration and duration (Sunderman 1989). A study also found that the chromosomal damage induced by in vitro exposure of Chinese Hamster Ovary (CHO) cells to carcinogenic nickel compounds preferably occurs in the heterochromatic regions, which contain more proteins and exist in a higher-condensed state. These findings could be explained by the high binding affinity of nickel ions for protein and the low affinity for DNA (Sen and Costa 1986). Most nickel ions in the cell nucleus preferentially interact with the histone, other than DNA, indicating the significance of nickel–protein interaction in the mechanism of nickel carcinogenesis. On the other hand, studies on the direct effect of nickel ions on DNA found that nickel ions could enhance the oxidation, hydroxylation and deglycosylation.

Of DNA bases (deoxynucleosides and deoxynucleotides) induced by active oxygen species (Datta et al. 1991; Littlefield et al. 1991). The most common DNA damage that occurs following the binding of DNA or protein with nickel includes: DNA strand breaks and DNA–protein, and DNA-interstrand crosslinks. The nickel compounds that have been found to be carcinogenic in experimental animals include metallic nickel, nickel monoxides, nickel hydroxides and crystalline nickel sulfides (Boffetta 1993).

### Other elements

There are other elements that have been classified as carcinogenic both in humans and experimental animals. The role of zinc as a carcinogenic agent is still subject to controversy. Zinc binding may be related to carcinogenesis since the metal does affect catabolic or anabolic enzymes. The role of zinc in RNA and DNA polymerase, its inhibitory effects on phosphodiesterase, and its activating role on membrane bound adenyl cyclase all points to the role of zinc in carcinogenesis (Schwartz 1975). While some studies have reported growth of local tumors at the site of injection of copper chloride and copper sulfate in experimental animals, negative results with metallic copper, copper acetate and copper sulfide have been reported (Boffetta 1993). Copper is believed to be the switch that turns on the angiogenesis process in tumor cells. Abnormally high serum copper levels are found in patients with many types of progressive tumors, making copper an obligatory cofactor in angiogenesis process. Copper-binding molecules (ceruloplasmin, heparin, and tripeptide glycly-histadyl-lysine) are non-angiogenic when free of copper, but they become angiogenic when bound to copper. As such, anti-copper drugs have been used cancer treatment (Brem et al. 1990). Cisplastin which is a platinum compound is known to cause leukemia in rats and lung and skin tumors in mice (IARC 1993). Selenium is an anti-carcinogen. Some selenoproteins show that they are relevant in scavenging ROS and reducing oxidative damage, while at times, there has been a strong inverse association between high serum selenium levels and pancreatic cancer risks.

### The way forward

The mechanistic effects of trace elements in carcinogenesis cannot be fully understood without further exploring the critical genetic events across the spectrum of human tumors with adequate sensitivity and scalability. To this end, new technologies have been developed in an attempt to define the spectrum of mutations, expression changes and other factors that are related to trace element carcinogenicity. Some of these technologies include:

*1. High throughput or new generation sequencing* Due to high demand for revolutionary DNA sequencing technologies that can deliver fast, inexpensive and accurate genome information, the recent years have seen a shift from the application of automated Sanger sequencing for genome analysis to a newer methods referred to as next-generation sequencing (NGS). The NGS technologies are not only cheap but also capable of producing enormous volume of data—sometimes in excess of one billion short reads per instrument run. The NGS technologies constitute various strategies that rely on a combination of template preparation, sequencing and imaging, and genome alignment and assembly methods. The broadest application of NGS has been the re-sequencing of human genomes, to enhance the understanding of how genetic differences affect health and disease.

Studies have been done using high-throughput genotyping to query over 238 known oncogene mutations across human tumor samples establishing a robust mutation distributions spanning over 17 cancer types (Roman et al. 2007). Of the 17 oncogenes analyzed, the study found 14 to be mutated at least once and that 30 % samples carried at least one mutation in addition to identifying a previously unrecognized oncogene mutations in several tumor types as well as observing an unexpectedly high number of co-occurring mutations. These results shone a new light in tumor genetics, where mutations involving multiple cancer genes may be interrogated simultaneously and in ‘real time’ to guide cancer classification and rational therapeutic intervention. High throughput sequencing also provides an alternative splicing complexity in human tissues using mRNA-Seq data, which provides more reliable measurements for exon inclusion levels. New splice junctions, many of which are tissue-specific, can be detected in approximately 20 % of multiexon genes (Qun et al. 2008). Targeted RNA-Seq can also combine NGS with capture of sequences from a relevant subset of a transcriptome through testing by capturing sequences from a tumor cDNA library with hybridization to oligonucleotide when probing specific cancer-related genes (Joshua et al. 2009). This method showed high selectivity, improved mutation detection enabling discovery of novel chimeric transcripts and provided RNA expression data.

*2. Gene expression profiling* Gene expression profiling is the measurement of the activity (the expression) of thousands of genes at once, by differentiating cells that are actively dividing, or showing how the cells react to a particular treatment to create a global picture of cellular function. Expression profiling experiments involves measuring the relative amount of mRNA expressed in two or more experimental conditions since altered levels of a specific sequence of mRNA suggest a changed need for the protein coded for by the mRNA, which may probably indicate a homeostatic response or a pathological condition. For instance, if a given cancer cells express higher levels of mRNA associated with a specific transmembrane receptor (specialized integral membrane proteins that take part in communication between the cell and the outside world) than normal cells do, it might signal that this receptor plays a role in that specific cancer type.

The detection of circulating tumor cells (CTCs) in the peripheral blood and microarray gene expression profiling of the primary tumor are two promising new technologies that have been shown to provide valuable prognostic profile data for patients with breast cancer (Molloy et al. 2012). The study was able to develop a unique microarray signature that can accurately predict CTC status in breast cancer patients based on gene expression in the primary tumor, which was not only independent of other clinical variables, but also MammaPrint, a prognostic microarray test used in the clinic for breast cancer patients (Molloy et al. 2012). Another study also used gene expression profiling for ER+ breast cancer prognosis among patients. Four microarray datasets were combined and research-based versions of PAM50 intrinsic subtyping and risk of relapse (PAM50-ROR) score, 21-gene recurrence score (OncotypeDX), Mammaprint, Rotterdam 76 gene, index of sensitivity to endocrine therapy (SET) and an estrogen-induced gene set were evaluated. All signatures were prognostic in patients with ER+ node-negative tumors, whereas most were prognostic in ER+ node-positive disease (Prat et al. 2012).

*3. Proteomics* Proteomics is the large-scale study of the identity, structural roles and biochemical functions of proteins, a vital part of living organism which is the main components of the physiological metabolic pathway of cell. Proteomics uses various biomarkers for disease diagnosis. The Proteomics techniques, which allow to test for proteins produced during a particular disease, hence helping to diagnose the disease quickly includes: western blot, immunohistochemical staining, enzyme linked immunosorbent assay (ELISA) also known as mass spectroscopy and secretomics (Klopfleisch et al. 2010; Klopfleisch and Gruber 2009). Researchers hope that proteomics research is inclined to help find new ways to diagnose cancer early (key to successful treatment), identify the best treatments for individual patients with specific types of cancer and determine recurrence of cancer after treatment.

The mass spectrometry (MS)-based quantitative proteomics is powerful tool for discovering disease biomarkers that can provide diagnostic, prognostic and therapeutic targets, in addition to addressing important problems in clinical and translational medical research (Liang et al. 2012). The MS-based quantification strategy and technical advances of several main quantitative assays includes two-dimensional (2D) gel-based methods, stable isotope labeling with amino acids in cell culture (SILAC), isotope-coded affinity tag (ICAT), the isobaric tags for relative and absolute quantification (iTRAQ), 18O labeling, absolute quantitation and label-free quantitation. They have been widely applied in identification of differential expression of proteins, post-translational modifications and protein–protein interactions in order to look for novel candidate cancer biomarkers from different physiological states of cells, body fluids or tissue samples (Liang et al. 2012).

## Conclusion

Since most of the elements that are carcinogenic are either naturally or through human activity present in the environment, awareness through public health information is necessary to reduce possible exposure that can affect thousands of human health. This information is also essential to create awareness and reduce occupational exposures to large populations that work directly in related industries as well as highlighting the effects of habits like smoking (both active and passive), which exposes individuals to more carcinogenic trace element cadmium. This review helps outline the main carcinogenic elements, their sources and exposure means and their carcinogenic effects which are essential for preventive purposes. More epidemiological data is necessary to understand the carcinogenic and anti-carcinogenic effects of these elements. Since certain cancer types vary with race, age group, organ, sex, heredity and environmental factors, generalization is not possible all the time. The use of the more advanced technologies some of which are discussed in the way forward section with the ability to analyze and compare large data quantities in real time can enhance the prognostic roles of trace elements among the global genetically and environmentally diverse populations with reliable accuracy. For instance, both gene expression profiling and proteomics have the potential diagnosis and identification of the best treatments for individual patients with specific types of cancer.

## References

[CR1] Agency for Toxic Substance and Disease registry (ATSDR) (2003) Toxicological profile for arsenic. U.S. Department of Health and Human Services, Public Health Humans Services, Center for Disease Control, Atlanta

[CR2] Agency for Toxic Substances and Disease Registry (ATSDR) (1998) Toxicological profile for chromium. U.S. Public Health Service, U.S. Department of Health and Human Services, Atlanta, GA

[CR3] Agency for Toxic Substances and Disease Registry (ATSDR) (2008) Public health statement. Beryllium. Cas #: 7440-41-7. Buford Highway, Atlanta, GA, USA, pp 1–7.a

[CR4] Ahsan H, Chen Y, Kibriya MG (2003). Susceptibility to arsenic-induced hyperkeratosis and oxidative stress genes myeloperoxidase and catalase. Cancer Lett.

[CR5] Ahsan H, Chen Y, Wa Q (2003). DNA repair gene XPD and susceptibility to arsenic-induced hyperkeratosis. Toxicol Lett.

[CR6] Allen BC, Crump KS, Shipp AM (1988). Correlation between carcinogenic potency of chemicals in animals and humans. Risk Anal.

[CR7] Ames B, Shigenaga K (1992). Oxidative stress is a major contribution to aging. Ann NY Acad Sci.

[CR8] Anil A, Puneet K, Dalim KB (2011). Trace elements in critical illness. J Endocrinol Metab.

[CR9] Augusto CM, Rhian MT (2011). Reactive oxygen species and endothelial function—role of nitric oxide synthase uncoupling and NOX family nicotinamide adenine dinucleotide phosphate oxidases. Basic Clin Phamacol Toxicol.

[CR10] Auld DS (2001) Zinc coordination sphere in biochemical zinc sites. Biometals 14(3–4):271–313 10.1023/a:1012976615056 11831461

[CR11] Bertini I, Gray BH, Lippard JS (1998) Valentine. In Bioinorganic chemistry. Viva Books Private Ltd., p 513

[CR12] Binner WF, Bridges WJ, Rose J (eds) (1983) Trace elements in health. Butterworth, p 1

[CR13] Boffetta P (1993). Carcinogenicity of trace elements with reference to evaluations made by the International Agency Research on cancer. Scand J Work Environ Health.

[CR14] Brem S, Zagzag D, Tsanaclis AM (1990). Inhibition of angiogenesis and tumor growth in the brain. Suppression of endothelial cell turnover by penicillamine and the depletion of copper, an angiogenic cofactor. Am J Pathol.

[CR15] Castro-González MI, Méndez-Armenta M (2008). Heavy metals: implications associated to fish consumption. Environ Toxicol Pharmacol.

[CR16] Chakraborti D, Sengupta MK, Rahaman MM, Amides Chowdhury UK, Hossain MA (2004). Ground water arsenic contamination and its health effects in the Ganga-Megna-Brahmaputra Plain. J Environ Monit.

[CR17] Chen Y, Graziano JH, Parvez F (2006). Modification of risk of arsenic-induced skin lesions by sunlight exposure, smoking, and occupational exposures in Bangladesh. Epidemiology.

[CR18] Cooper GR, Harrison PA (2009). The uses and adverse effects of beryllium on health. Indian J Occup Environ Med.

[CR19] Dara SS (1997). Text book of environmental chemistry and its pollution control.

[CR20] Datta AK, Riggs CW, Fwvash MJ, Kasprzak KS (1991). Mechanisms of nickel carcinogenesis. Interaction of Ni (II) with 2′-deoxynucleosides and 2′-deoxynucleotides. Chem Biol Interact.

[CR21] Dobis DR, Sawyer RT, Gillespie MM, Huang J, Newman LS, Maier LA (2008). Modulation of lymphocyte proliferation by antioxidants in chronic beryllium disease. Am J Respir Crit Care Med.

[CR22] Figueroa E (2008). Are more restrictive food cadmium standards justifiable health safety measures or opportunistic barriers to trade? An answer from economics and public health. Sci Total Environ.

[CR23] Fontenot AP, Falta MT, Freed BM, Newman LS, Kotzin BL (1999). Identification of pathogenic T cells in patients with beryllium-induced lung disease. J Immunol.

[CR24] Fontenot AP, Canavera SJ, Gharavi L, Newman LS, Kotzin BL (2002). Target organ localization of memory CD4+ T cells in patients with chronic beryllium disease. J Clin Invest.

[CR25] Giaginis C, Gatzidou E, Theocharis S (2006). DNA repair systems as targets of cadmium toxicity. Toxicol Appl Pharmacol.

[CR26] Goodman G, Wilson R (1991). Quantitative prediction of human cancer risk from rodent carcinogenic potencies: a closer look at the epidemiological evidence for some chemicals not definitively carcinogenic in humans. Regulat Pharmacol Toxicol.

[CR27] Hainaut P, Milner J (1993). A structural role for metal ions in the “wild type” confirmation of the tumor suppressor protein p53. Cancer Res.

[CR28] Harrison N (2001) Inorganic contaminants in food. In: Watson DH (ed) Food chemical safety contaminants, 1st edn. Woodhead Publishing Ltd, Cambridge, pp 148–168. ISBN 1-85573-462-1

[CR29] Hartwig A, Snyder RD, Schlepegrell R (1990). Modulation by Co (II) of UV-induced DNA repairs, mutagenesis and sister chromatid exchanges in mammalian cells. Mutat Res.

[CR102] Hartwig A, Snyder RD, Schlepegrell R, Beyersmann D (1991) Modulation by Co(II) of UV-induced DNA repair, mutagenesis and sister chromatid exchanges in mammalian cells. Mutat Res 248:177–185 10.1016/0027-5107(91)90099-a 2030707

[CR30] Henle ES, Linn S (1997). Formation, prevention, and repair of DNA damage by iron/hydrogen peroxide. J Biol Chem.

[CR31] Ho E (2004) Zinc deficiency, DNA damage and cancer risk. J Nutr Biochem 15:572–578 10.1016/j.jnutbio.2004.07.005 15542347

[CR32] Hollstein M, Rice K, Greenblatt SM, Soussi T, Fuchs R, Sorlie T, Hovig E, Smith-Sorensen B, Montesano R, Harris CC (1994) Database of p53 gene somatic mutations in human tumors and cell lines. Nucleic Acids Res 22:3551–3555 PMC308317 7937055

[CR33] Huff J (1999). Long-term chemical carcinogenesis bioassays predict human cancer hazards. Issues, controversies, and uncertainties. Ann NY Acad Sci.

[CR34] Huff J (1999b) Value, validity, and historical development of carcinogenesis studies for predicting and confirming carcinogenic risks to humans. In: Kitchin KT (ed) Carcinogenicity. Testing, predicting, and interpreting chemical effects.Marcel Dekker, Inc., New York, pp 21–123

[CR104] International Agency for Research on Cancer (IARC) (1980) IARC monographs on the evaluation of carcinogenic risks to humans. Arsenic and arsenic compounds, vol 23, pp 39

[CR105] International Agency for Research on Cancer (IARC) (1987) IARC monographs on the evaluation of carcinogenic risks to humans. Overall evaluation of carcinogenicity, an updating of IARC monographs: lead and lead compounds, vol 1–42 (suppl 7), pp 230

[CR35] International Agency for Research on Cancer (IARC) (1989) Antimony trioxide and antimony trisulfide. IARC, Lyon, pp 291–305 (IARC monographs on the evaluation of carcinogenic risks to humans; vol 47) PMC7682321 2699902

[CR36] International Agency for Research on Cancer (IARC) (1991) Cobalt and cobalt compounds.IARC, Lyon, pp 363–472 (IARC Monograms on the evaluation of carcinogenic risks to humans; vol 52) PMC7682369 1960848

[CR37] International Agency for Research on Cancer (IARC) (1993). IARC monographs on the evaluation of carcinogenic risks to humans; vols 1–58.

[CR103] International Agency for Research on Cancer (IARC) (1997) IARC monographs on the evaluation of carcinogenic risks to humans, vol 87. IARC, Lyon, pp 1–43 PMC7681469 1683674

[CR38] Joshua ZL, Michael FB, Xian A, Peter R, Alexandre M, Timothy F, Chad N, Levi AG, Andreas G (2009). Targeted next-generation sequencing of a cancer transcriptome enhances detection of sequence variants and novel fusion transcripts. Genome Biol.

[CR39] Klopfleisch R, Gruber AD (2009). Increased expression of BRCA2 and RAD51 in lymph node metastases of canine mammary adenocarcinomas. Vet Pathol.

[CR40] Klopfleisch R, Klose P, Weise C, Bondzio A, Multhaup G, Einspanier R, Gruber AD (2010). Proteome of metastatic canine mammary carcinomas: similarities to and differences from human breast cancer. J Proteome Res.

[CR41] Landolph J (1994). Molecular mechanisms of transformation of CH3/10T1/2 C1 8 mouse embryo cells and diploid human fibroblasts by carcinogenic metal compounds. Environ Health Perspect.

[CR42] Leonard A, Lauwerys R (1987). Mutagenicity, carcinogenicity and tetragenicity of beryllium. Mutat Res.

[CR43] Liang S, Xu Z, Xu X, Zhao X, Huang C, Wei Y (2012) Quantitative proteomics for cancer biomarker discovery, combinatorial chemistry and high throughput screening. Benthan Sci 15(3):221–231 10.2174/138620712799218635 22221055

[CR44] Littlefield NA, Fullerton FR, Pbirier LA (1991). Hydroxylation and deglycosylation of 2′-deoxyguanosine in the presence of magnesium and nickel. Chem Biol Interact.

[CR45] Lloyd DR, Phillips DH, Carmichael PL (1997). Generation of putative intrastrand cross-links and strand breaks in DNA by transition metal ion-mediated oxygen radical attack. Chem Res Toxicol.

[CR46] Mao Y, Liu KJ, Jiang JJ (1996). Generation of reactive oxygen species by Co (II) from H2O2 in the presence of chelators in relation to DNA damage and 2′-deoxyguanosine hydroxylation. J Toxicol Environ Health.

[CR47] Maurici D, Aardema M, Corvi R (2005). Genotoxicity and mutagenicity. Altern Lab Anim.

[CR48] Mehmet Y (2006). Comprehensive comparison of trace metal concentrations in cancerous and non-cancerous human tissues. Curr Med Chem.

[CR49] Ming-Ho Y (2005) Environmental toxicology: biological and health effects of pollutants, chap 12, 2nd edn. CRC Press LLC, Boca Raton, USA, ISBN 1-56670-670-2

[CR50] Molloy TJ, Roepman P, Naume B, van’t Veer LJ (2012). A prognostic gene expression profile that predicts circulating tumor cell presence in breast cancer patients. PLoS ONE.

[CR51] Mouron SA, Golijow CD, Dulout FN (2001). DNA damage by cadmium and arsenic salts assessed by the single cell gel electrophoresis assay. Mutat Res.

[CR52] Mudhoo A, Sharma SK, Garg VK, Tseng T-H (2001). Arsenic: an overview of applications, health and environmental concerns and removal process. Crit Rev Environ Sci Technol.

[CR53] Nackerdien Z, Kasprzak KS, Rao G (1991). Nickel (II) and cobalt (II) dependent damage by hydrogen peroxide to the DNA bases in isolated human chromatin. Cancer Res.

[CR54] Nordberg G, Alessio L, Herber RM (ed) (1992) Cadmium in the human environment: toxicity and carcinogenicity. IARC (Scientific publication; no. 118), Lyon 1303931

[CR55] Nzengue Y, Steiman R, Garrel C, Lefèbvre E, Guiraud P (2008). Oxidative stress and DNA damage induced by cadmium in the human keratinocyte HaCaT cell line: role of glutathione in the resistance to cadmium. Toxicology.

[CR56] OECD (2006) Detailed review paper on cell transformation assays for detection of chemical carcinogens. DRP No. 31. Fourth draft version 10.1016/j.mrgentox.2011.11.007 22120692

[CR57] Palmer BE, Mack DG, Martin AK, Gillespie M, Mroz MM, Maier LA (2008). Up-regulation of programmed death-1 expression on beryllium specific CD4+ T cells in chronic beryllium disease. J Immunol.

[CR58] Prat A, Parker SJ, Fan C, Chiang MC, Miller LD, Bergh J, Chia SK, Benard PS, Nielson TO, Ellis MJ, Carey LA, Perou CM (2012). Concordance among gene expression-based predictors for ER-positive breast cancer treated with adjuvant tamoxifen. Ann Oncol.

[CR59] Pu YS, Jan KY, Wang TC (2007). 8-Oxoguanine DNA glycosylase and MutY homolog are involved in the incision of arsenite-induced DNA adducts. Toxicol Sci.

[CR60] Quintanilla-Vega B, Hoover DJ, Silbergeld EK, Waalkes M, Anderson LD (1997) Interaction between lead and protamine 2 from human spermatozoa. International symposia on environment, lifestyle, and fertility, Aarhus, Denmark

[CR61] Qun P, Ofer S, Leo JL, Brendan JF, Benjamin JB (2008). Deep surveying of alternative splicing complexity in the human transcriptome by high-throughput sequencing. Nat Genet.

[CR62] Reddy BS, Charles JM, Raju NJG, Vijayan V, Reddy SB, Kumar RM, Sundareswar B (2003). Trace elemental analysis of carcinoma kidney and stomach by PIXE method. Nucl Instr Methods Phys Res B.

[CR63] Reichard JF, Puga A (2010). Effects of arsenic exposure on DNA methylation and epigenetic gene regulation. Epigenomics.

[CR64] Roman KT, Alissa CB, Ralph MD, Wendy W, Thomas L, William ML, Meng W, Whei F, Thomas Z, Laura EM, Jeffrey CL, Rick N, Charlie H, Mary G (2007). High-throughput oncogene mutation profiling in human cancer. Nat Genet.

[CR65] Sakurai T, Kojima C, Ohta T, Fujiwara K (2004). Evaluation of in vivo acute immunotoxicity of major organic arsenic compound arsenobetaine in seafood. Int Immunopharmacol.

[CR66] Sarkar B (1995). Metal replacement in DNA-binding zinc finger proteins and its relevance to mutagenicity and carcinogenicity through free radical generation. Nutrition.

[CR67] Sawyer CN, McCarty PL, Parkin GF (2003) Chemistry for environmental and engineering science, 5th edn. Mc Graw Hill, NY, ISBN 0-07-248066-1

[CR106] Schwartz KM (1975) Role of trace elements in cancer. Cancer Res 35:3481–3487 1104155

[CR68] Second Annual Report (1981) Beryllium (CAS No. 7440-41-7) and beryllium compounds. Substance profiles. Report on carcinogens, 1st edn, Georgia, USA, pp 1–3

[CR69] Sen P, Costa M (1986). Incidence and localization of sister chromatic exchange induced by nickel and chromium compounds. Carcinogenesis.

[CR100] Steenland K, Ward E (1991) Lung cancer incidence among patients with beryllium disease: a cohort mortality study. J Natl Can Ist 83:1380–1385 10.1093/jnci/83.19.1380 1920480

[CR101] Stiefel TK, Schultz K, Zorn H, Tolg G (1980) Toxicodynamic studies of beryllium. Arch Toxicol 45(2):81–92 10.1007/BF01270905 6162434

[CR70] Stoeckle JD, Mancuso T (1974). Beryllium disease. Science.

[CR71] Sunderman FW (1989). Mechanism of nickel carcinogenesis. Scand J Work Environ Health.

[CR72] Uneyama C, Toda M, Yamamoto M, Morikawa K (2007). Arsenic in various foods: cumulative data. Food Addit Contam.

[CR73] United Nations Economic Commission for Europe (UNECE) (2004) Globally harmonized system of classification and labeling of chemicals (GHS). Part 3, Health and environmental hazards, chap 3.6, carcinogenicity

[CR74] United States Environmental Protection Agency (USEPA) (2005) Arsenic in drinking water fact sheet

[CR75] U.S. Environmental Protection Agency (USEPA) (1998) Toxicological review of trivalent chromium. National Center for Environmental Assessment, Office of Research and Development, Washington, DC

[CR76] Valco M, Morris H, Cronin MTD (2005). Current. Med Chem.

[CR77] Waisberg M, Joseph P, Hale B, Beyersmann D (2003). Molecular and cellular mechanisms of cadmium carcinogenesis. Toxicology.

[CR78] Wang S, Leonard SS, Ye J, Ding M, Shi X (2000) The role of hydroxyl radical as a messenger in Cr(VI)-induced p53 activation. Am J Physiol Cell Physiol 279(3):C868–C875 10.1152/ajpcell.2000.279.3.C868 10942736

[CR79] Wang Z, Farris GM, Newman LS, Shou Y, Maier LA, Smith HN (2001). Beryllium sensitivity is linked to HLA-DP genotype. Toxicology.

[CR80] Wang Y, Fang J, Leonard SS, Rao KM (2004). Cadmium inhibits the electron transfer chain and induces reactive oxygen species. Free Radic Biol Med.

[CR81] WHO (2004a) Guidelines for drinking-water quality. Sixty-first meeting, Rome, 10–19 June 2003. Joint FAO/WHO Expert Committee on Food Additives

[CR82] WHO (2004b) Evaluation of certain food additives contaminants. Sixty-first report of the joint FAO/WHO Expert Committee on Food Additives. WHO Technical Report Series, No. 922

[CR83] WHO (2006) Evaluation of certain food contaminants. Sixty-fourth report of the Joint FAO/WHO Expert Committee on Food Additives. WHO Technical Report Series, No. 930

[CR84] Yurifuji T, Tsuda T, Doi H (2011). Cancer excess after arsenic exposure from contaminated milk powder. Environ Health Prev Med.

